# Serum interleukin-6 is an indicator for severity in 901 patients with SARS-CoV-2 infection: a cohort study

**DOI:** 10.1186/s12967-020-02571-x

**Published:** 2020-10-29

**Authors:** Jing Zhang, Yiqun Hao, Wuling Ou, Fei Ming, Gai Liang, Yu Qian, Qian Cai, Shuang Dong, Sheng Hu, Weida Wang, Shaozhong Wei

**Affiliations:** 1 Department of Medical Oncology, Hubei Cancer Hospital, Tongji Medical College, Huazhong University of Science and Technology, Wuhan, 430079 Hubei China; 2 Department of Rheumatology and Immunology, The First Hospital of Shanxi Medical University, Taiyuan, 030001 Shanxi China; 3 Department of Thoracic Surgery, Hubei Cancer Hospital, Tongji Medical College, Huazhong University of Science and Technology, Wuhan, 430079 Hubei China; 4 Department of Radiation Oncology, Hubei Cancer Hospital, Tongji Medical College, Huazhong University of Science and Technology, Wuhan, 430079 Hubei China; 5 Department of Hematological Oncology, State Key Laboratory of Oncology in South China, Collaborative Innovation Center for Cancer Medicine, Sun Yat-Sen University Cancer Center, No. 651 Dongfeng East Road, Yuexiu District, Guangzhou, 510060 Guangdong China; 6 Department of Gastrointestinal Surgery & Colorectal Cancer Center of Hubei Province, Hubei Cancer Hospital, Tongji Medical College, Huazhong University of Science and Technology, No. 116 Zhuodaoquan South Road, Hongshan District, Wuhan, 430079 Hubei China

**Keywords:** SARS-CoV-2, COVID-19, Interleukin-6, Tocilizumab

## Abstract

**Background:**

Interleukin-6 (IL-6) was proposed to be associated with the severity of coronavirus disease 2019 (COVID-19). The present study aimed to explore the kinetics of IL-6 levels, validate this association in COVID-19 patients, and report preliminary data on the efficacy of IL-6 receptor blockade.

**Methods:**

We conducted a retrospective single-institutional study of 901 consecutive confirmed cases. Serum IL-6 concentrations were tested on admission and/or during hospital stay. Tocilizumab was given to 16 patients with elevated IL-6 concentration.

**Results:**

366 patients were defined as common cases, 411 patients as severe, and 124 patients as critical according to the Chinese guideline on diagnosis and treatment of COVID-19. The median concentration of IL-6 was < 1.5 pg/ml (IQR < 1.50–2.15), 1.85 pg/ml (IQR < 1.50–5.21), and 21.55 pg/ml (IQR 6.47–94.66) for the common, severe, and critical groups respectively (*P* < 0.001). The follow-up kinetics revealed serum IL-6 remained high in critical patients even when cured. An IL-6 concentration higher than 37.65 pg/ml was predictive of in-hospital death (AUC 0.97 [95% CI 0.95–0.99], *P* < 0.001) with a sensitivity of 91.7% and a specificity of 95.7%. In the 16 patients who received tocilizumab, IL-6 concentrations were significantly increased after administration, and survival outcome was not significantly different from that of propensity-score matched counterparts (n = 53, *P* = 0.12).

**Conclusion:**

Serum IL-6 should be included in diagnostic work-up to stratify disease severity, but the benefit of tocilizumab needs further confirmation.

*Trial registration* retrospectively registered.

## Introduction

As of the 4th April 2020, the spread of severe acute respiratory syndrome coronavirus-2 (SARS-CoV-2) still shows no signs of slowing down with 75,853 new cases confirmed globally everyday [1]. The infection caused by this novel virus was named coronavirus disease 2019 (COVID-19) by the World Health Organization (WHO). According to the report of the WHO-China Joint Mission, in the first 2 months of the epidemic in China, 13.8% of the patients developed severe disease requiring oxygen therapy and 6.1% developed to a critically ill stage requiring intensive care [2]. These critically ill patients have an inferior prognosis, with a case-fatality rate of up to 50% [3]. If they recover the average length of hospital stay is up to 50 days, putting stress on the capacity of intensive care units in epidemic areas and in turn increasing mortality rates [4]. To date, no specific anti-viral approach has proven successful, and the mainstay of clinical management is largely treatment of the symptoms. Early detection and treatment for severe and critically ill patients are crucial issues requiring urgent investigation.

By summarizing the clinical characteristics, abnormal immunologic features were identified among COVID-19 patients with a higher serum concentration of proinflammatory cytokines [5]. Serum concentrations of interleukin 2 receptor (IL-2R), interleukin 6 (IL-6), interleukin 8 (IL-8), interleukin 10 (IL-10), and tumor necrosis factor α (TNFα) were significantly higher in deceased patients than in recovered counterparts [6]. In addition, the levels of these cytokines were markedly higher in severe cases compared with moderate cases [7], which suggests the necessity of IL-6 detection for early prediction of severity [8]. It is postulated that host-directed therapies aiming at ameliorating excessive and aberrant host immune responses might be effective [9]. Tocilizumab, a monoclonal antibody of IL-6 receptor, is a potential therapeutic option. Trials were conducted to investigate the efficacy and safety of tocilizumab in severe COVID-19 patients (ChiCTR2000029765, ChiCTR2000030894, NCT04317092, NCT04332913, NCT04320615, NCT04306705, NCT04332094, NCT04331808, NCT04315480, NCT04333914, NCT04331795). Preliminary results indicated that tocilizumab could reduce C-reactive protein, improve clinical symptoms and prognosis [10]. The purpose of the present study is to describe the distribution of baseline IL-6 levels and its kinetics among different stratifications of COVID-19 patients in Wuhan, and the outcome of compassionate use of tocilizumab.

## Methods

### Study population, setting and data collection

Patients with SARS-CoV-2 infection who were admitted to Leishenshan Hospital in Wuhan, one of the designated temporary hospitals for COVID-19 in the epicenter since February 5, 2020 were included in the current study. The diagnostic criteria for COVID-19 followed the interim or 7th edition guideline of The National Health Commission [11]. which mainly included epidemiological history, clinical symptom, thoracic CT examination, and results of SARS-CoV-2 nucleic acid detection. The enrollment criterion was that serum IL-6 was detected at least one time in patients with SARS-CoV-2 infection. Serum IL-6 level was first tested as a cross section examination among patients (Late February, 2020), and then routinely tested at admission and in patients with previously elevated IL-6.

901 patients were finally identified and included in this study. As an observational retrospective study, no exclusion criteria were set. Demographic data, information on comorbidities, laboratory results during admission and/or hospital stay, and outcome followed-up to the 2nd April 2020 were extracted from electric medical records through the built-in information system (DTHealth, Donghua healthcare, Inc.). Patient’s names and IDs were deleted, and researchers analyzed only renumbered anonymous data. Meanwhile, cured patients but with other coexisting disorders that need to be treated might be transferred to another hospital for further treatment.

### Laboratory measurements

Real time reverse transcription polymerase chain reaction assay (RT-PCR) for SARS-Cov-2 was used for detection in throat or nasal swab specimens. Wuhan Kindstar Diagnostic Laboratory (Kindstar Global Technology, Inc. Beijing, China) was responsible for the diagnosis using 2019-nCoV detection kit (DA0940, Daan Gene Co., Ltd. Guangzhou, China). The laboratory has been authorized by the Chinese Center for Disease Control and Prevention, and the reliability of its methodology and quality control is guaranteed. Serum IL-6 assays were determined in plasma samples by electro-chemiluminescence immunoassay using Roche Cobas e411 (Roche Diagnostics GmbH, Mannheim, Germany). The lower detection limit of this kit is 1.5 pg/ml, and the upper detection limit is 5000 pg/ml without dilution. The normal upper limit is 7 pg/ml.

### Treatments

Patients received treatment in accordance with the interim or 7th edition guideline of The National Health Commission, China [11]. All patients received symptomatic support treatment and close monitoring of vital signs. High-flow nasal cannula oxygen therapy, non-invasive positive pressure ventilation, invasive mechanical ventilation, and other respiratory support were provided to appropriate patients dependent on their individual situations. Renal replacement therapy included hemodialysis was also provided. Antiviral drugs including arbidol, oseltamivir, ribavirin, and favipiravir were prescribed in clinical trials or in the form of compassionate use. Chloroquine and vitamin C were tested as well in clinical trials. Corticosteroids were prescribed short-term when respiratory distress and/or cytokine storm occurred. Actemra (Tocilizumab, approximately 8 mg/kg, Chugai Pharmaceutical) was given to part of patients with elevated IL-6 level in the form of compassionate use.

### Study definitions

The stratifications of COVID-19 disease spectrum were in accordance with the interim or 7th edition guideline of The National Health Commission [11]. Mild cases were defined as paucisymptomatic with normal lung imaging. Common cases were defined as moderate symptoms with pneumonia manifestations on CT scan, but not consistent with severe cases. Severe cases were defined as any patient who met any of the following criteria: shortness of breath with respiratory rate ≥ 30 times/min, oxygen saturation ≤ 93% at rest, PaO_2_/oxygen concentration ≤ 300 mmHg. Moreover, patients with > 50% lesion progression within 1 to 2 days on thoracic CT scan were also categorized as severe cases. Critical cases were defined as any patient who met any of the following criteria: respiratory failure with support of mechanical ventilation; occurrence of shock; other organ failure requiring treatment in the ICU.

### Statistics

Descriptive statistics were applied to summarize the demographic data. Results are reported as medians and interquartile ranges or means with standard deviations or counts and frequency. One-way ANOVA was applied to detect significant differences among stratifications. Given the differences in clinical characteristics between patients who received tocilizumab and patients without it, propensity-score matching was used to identify a limited cohort with balanced characteristics. Statistical Package for Social Sciences (SPSS) 25.0 software (IBM, Armonk, NY, USA), Stata software (version 15.0) (StataCorp LLC, College Station, TX, USA), the R Project for Statistical Computing (R version 3.3.0) (The R Foundation, https://www.r-project.org), and GraphPad Prism (version 8.2.1) (GraphPad, Inc.) were used for statistical analysis and illustrations.

## Results

A total of 901 patients were enrolled in the present study. The demographic and clinical characteristics are shown in Table 1. Except for autoimmune diseases, malignancies, and administration of tocilizumab, all features were statistically different among the three distinct categories based on severity of disease. Patients were significantly older in the critical group with a median age of 68.5 years, compared with the severe (64.0 years) and common groups (54.0 years). The proportion of male patients increased significantly from 44% in the common group to 58.9% in the critical group. Patients with chronic medical conditions were more likely to be in a higher severity group, ranging from 46.7% in the common group to 75.0% in the critical group. The five most common comorbidities in the entire cohort were hypertension, diabetes mellitus, coronary heart disease, stroke, and cancer. Although the incidence of chronic kidney disease is not high among the entire cohort, patients suffering from this comorbidity were more likely to develop critical disease with 10/13 (76.9%) in the critical group.

**Table 1:** Clinical characteristics of patients at baseline

Parameters	Entire cohort (N = 901)	Common (n = 366)	Severe (n = 411)	Critical (n = 124)	*P* value
Age, years (median, IQR)	60.0 (49.0–69.0)	54.0 (42.8–62.0)	64.0 (52.0–71.0)	68.5 (58.0–78.0)	< 0.001
Sex-n, %					0.015
Male	435 (48.3)	161 (44.0)	201 (48.9)	73 (58.9)	
Female	466 (51.7)	205 (56.0)	210 (51.1)	51 (41.1)	
Coexisting disorders-n, %	509 (56.5)	171 (46.7)	245 (59.6)	93 (75.0)	< 0.001
Hypertension	289 (32.1)	82 (22.4)	149 (36.3)	58 (46.8)	< 0.001
Diabetes mellitus	126 (14.0)	38 (10.4)	57 (13.9)	31 (25.0)	< 0.001
Chronic obstructive lung disease	17 (1.9)	3 (0.8)	7 (1.7)	7 (5.6)	< 0.001
Hemorrhagic or ischemic stroke	38 (4.2)	6 (1.6)	15 (3.6)	17 (13.7)	< 0.001
History of tuberculosis	13 (1.4)	4 (1.1)	5 (1.2)	4 (3.2)	0.002
Autoimmune diseases	10 (1.1)	4 (1.1)	4 (1.0)	2 (1.6)	0.49
Chronic kidney disease	13 (1.4)	0 (0)	3 (0.7)	10 (8.1)	< 0.001
Malignancies	22 (2.4)	8 (2.2)	13 (3.2)	1 (0.8)	0.30
Coronary artery disease	73 (8.1)	18 (4.9)	37 (9.0)	18 (14.5)	0.002
Chronic dialysis	7 (0.8)	0 (0)	2 (0.5)	5 (4.0)	< 0.001
Baseline IL-6					< 0.001
Normal-n, %	708 (78.6)	347 (94.8)	329 (80.0)	32 (25.8)	
Elevated^a^-n, %	193 (21.4)	19 (5.2)	82 (20.0)	92 (74.2)	
Tocilizumab used-n, %	16 (1.8)	4 (1.1)	7 (1.7)	5 (4)	0.39
Outcomes^b^-n, %					< 0.001
Cured/discharged	683 (75.8)	290 (79.2)	340 (82.7)	53 (42.7)	< 0.001
Continued hospitalization	193 (21.4)	76 (20.8)	70 (17.0)	47 (37.9)	< 0.001
Death	24 (2.7)	0 (0)	0 (0)	24 (19.4)	< 0.001

^a^Higher than upper limit of normal

^b^1 missing data in the severe group

**Fig. 1:**
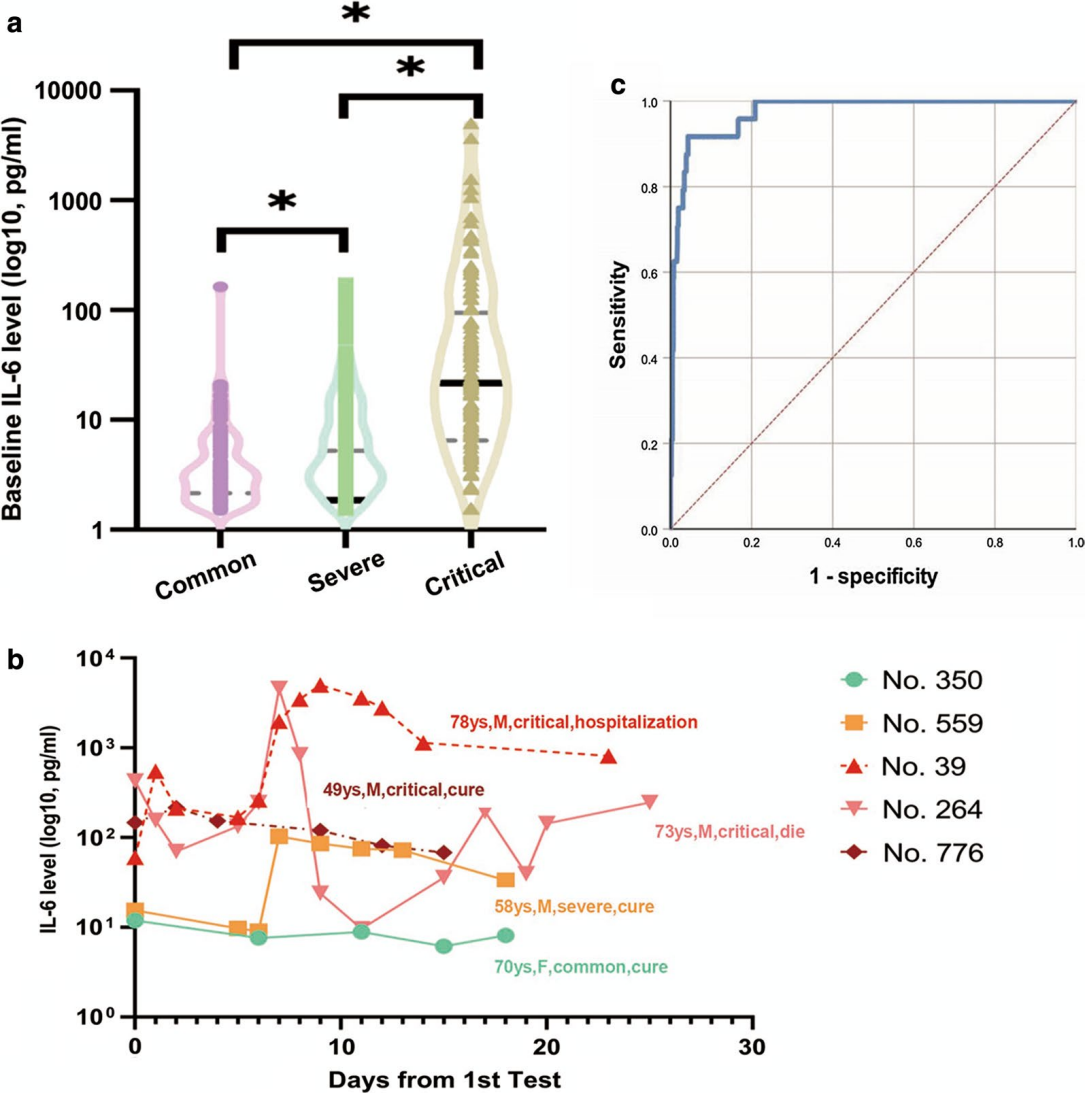
The distribution, survival prediction and kinetics of IL-6 in patients with SARS-CoV-2 infection. **a** The baseline level of serum IL-6 in 901 patients who were classified into three distinct prognostic subgroups (common, severe, and critical) based on clinical characteristics. **b** The typical dynamics of serum IL-6 in these three subgroups. *ys* years, *M* male, *F* female. Day 0: the day patient took the first IL-6 examination. * means *P* < 0.01. **c** Receiver operating characteristic curve was applied to identify the cut-off of IL-6 for prediction of in-hospital death in this cohort (N = 901) (*P* < 0.001)

With respect to the entire cohort, 21.4% patients had elevated baseline IL-6 concentrations. The percentage of patients with elevated IL-6 in the critical subgroup was as high as 74.2%, 3.5 and 14 times more than that in the severe and common subgroups, respectively (Table 1). Figure 1a demonstrates the serum IL-6 levels in different subgroups. The median concentration of IL-6 at baseline was < 1.5 pg/ml (IQR < 1.50–2.15, ranging from < 1.50 to 162.7), 1.85 pg/ml (IQR < 1.50–5.21, ranging from < 1.50 to 177.1), and 21.55 pg/ml (IQR 6.47–94.66, ranging from < 1.50 to > 5000) for the common, severe and critical groups, respectively (*P* < 0.001). Dynamic observation showed that IL-6 concentrations remained relatively higher among the critical subgroup even when cured (Fig. 1b). The kinetics of IL-6 concentrations in these three subsets is displayed in Additional file 1: Fig. S1.

As of the 2nd April 2020, 193/901 (21.4%) patients were still in hospital, including 76 common cases, 70 severe cases and 47 critical cases (*P* < 0.001). As expected, the cured/discharged rate is much higher in the non-critical subgroups (about 80%), which is about 2 times more than the critical subgroup (*P* < 0.001). A total of 24 deaths were observed in this cohort, and all these deaths occurred in the critical subgroups. However, the rates of continued hospitalization and cured/discharged as well as survival did not differ significantly between the common and severe groups (*P* = 0.3). The baseline IL-6 concentration was highly predictive of in-hospital death for COVID-19 patients (ROC AUC 0.97 [95% CI 0.95–0.99], Fig. 1c). The cut-off of IL-6 level was 37.65 pg/ml with a sensitivity of 91.7% and a specificity of 95.7% (the largest Youden’s Index = 0.873, *P* < 0.001).

**Fig. 2:**
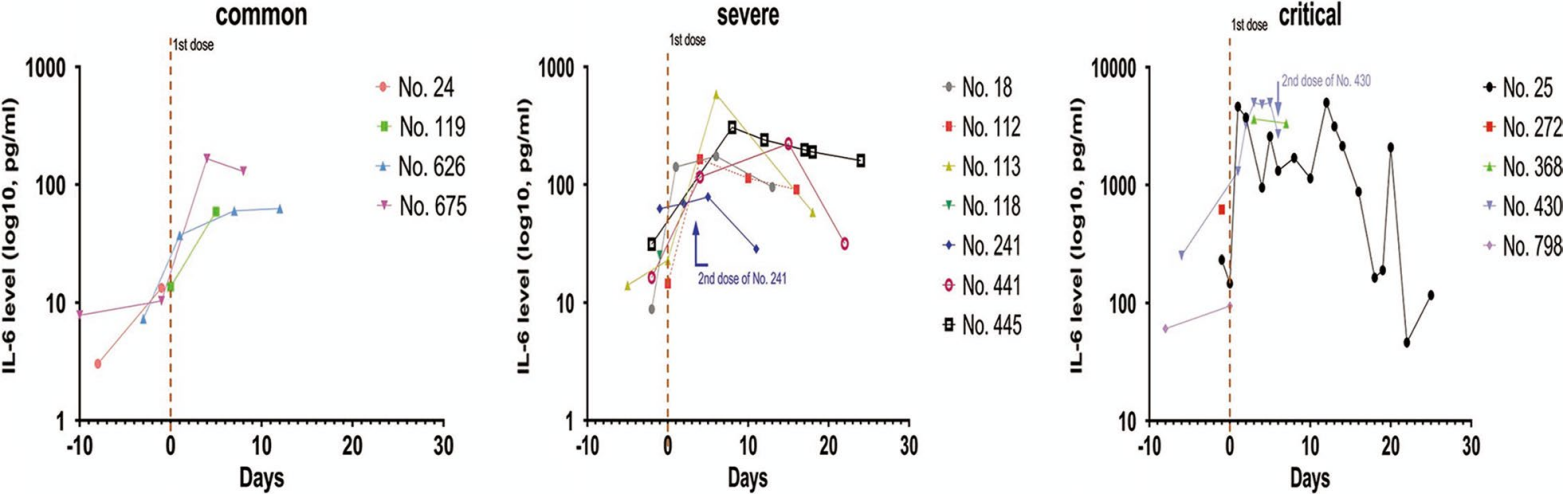
The dynamics of serum IL-6 in patients receiving tocilizumab (n = 16). Day 0: the day patient received the first dose of tocilizumab

**Fig. 3:**
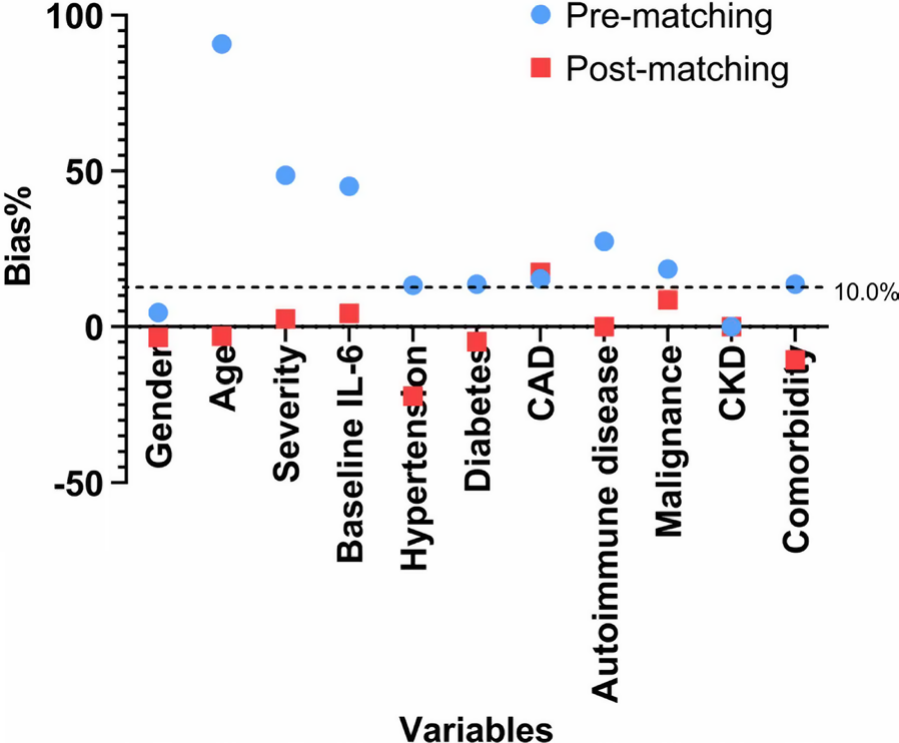
The parameters involved in the analysis of propensity-score matching and the pre-matching and post-matching bias. *CAD* coronary artery disease, *CKD* chronic kidney disease

Of 901 patients, 16 patients received tocilizumab treatment. Characteristics of patients receiving tocilizumab were summarized in Additional file 2: Table S1. IL-6 levels after tocilizumab administration were tested multiple times. Figure 2 shows the kinetics of IL-6 levels in these 16 patients. 2 patients received a second dose of monoclonal antibody. Almost all the patients experienced a sharp increase of IL-6 level immediately after administration of tocilizumab. For the short-term outcomes, there was no significant difference in patients receiving tocilizumab or not (15.4% deaths versus 7.5% deaths, relative ratio, 0.49, 95% CI 0.10–2.39, *P* = 0.4). Next, it was examined in a propensity-score matched cohort. Before propensity-score matching, several baseline variables greatly differed in patients receiving tocilizumab or not. In the cohort post-matching, 13 patients who received tocilizumab were matched with 53 patients who did not receive tocilizumab. The C-statistics for the model was 0.992. After matching, survival outcome was still not significantly differed from that of the propensity-score matched 53 counterparts (*P* = 0.12), and the standardized differences were less than 10.0% in 10 variables except coronary heart disease and hypertension, indicating that differences between the two groups were restricted to a small extent (Fig. 3).

## Discussion

This single-institutional case series describes 901 patients with SARS-CoV-2 infection and symptoms ranging from common to critically ill. Serum IL-6 concentration was tested and analyzed in all the patients. The IL-6 level was tested multiple times in patients with consistently high levels of IL-6 to obtain kinetics profiles. It is increasingly recognized that excessive, malfunctional host immune response may play an important role in the development and maintenance of critical stages of COVID-19. Some teams had reported IL-6 expression in COVID-19 patients, suggesting that elevated IL-6 and other cytokine levels correlated with severity of this disease, however, only a few patients (21–100) were enrolled in these studies [7, 12, 13]. Though our result was consistent with that of these studies, this large-sample study gives general profiles of baseline IL-6 distribution among patients with common, severe and critical subsets, suggesting a strong correlation between IL-6 level and severity of COVID-19.

However, correlation does not guarantee causation. Drastically elevated IL-6 levels (> 100 pg/ml), were closely associated with detectable serum SARS-CoV-2 viral load [14]. Nevertheless, the bridge between IL-6 and virus is yet to be built. A study related to SARS-CoV [15] revealed that anti-spike IgG abrogated the wound-healing response and promoted proinflammatory cytokines production (IL-8, IL-6, etc*.*), therefore mediated acute lung injury. This finding implied that IL-6 receptor blockade might be potential way to mitigate lung injury. Nevertheless, some issues are still to be addressed. Proinflammatory cytokines including IL-6 are universally elevated in sepsis and other infections. Zhao et al. [16] compared IL-6 levels between COVID-19 patients and other pneumonia patients, and found that they did not differ significantly (19.34 pg/ml versus 15.06 pg/ml, *P* = 0.7). In sepsis, these cytokines played a minor role for their short half-life [17]. Besides, IL-6 was shown to be elevated to over 1000 pg/ml in patients with sepsis, even higher than half of the critically ill COVID-19 patients in our cohort, but acute lung injury was not more common in sepsis than COVID-19. According to our kinetics results, we suggest that the duration alongside with the level of IL-6 elevation might play an important role in the severity of disease.

A systemic review conducted by Coomes et al*.* showed that IL-6 was associated with adverse clinical outcomes [18]. Moreover, some studies indicated that different IL-6 cut-off values showed distinct clinical significance. Yong et al. identified the cut-off value of 24.3 pg/ml of IL-6 combining with D-Dimer for early detection of severe cases in a cohort of 43 cases [8]. Giofoni et al. identified a cut-off value of 25 pg/ml of serum IL-6 as an independent risk factor of progression for severe COVID-19 and/or in-hospital mortality in a cohort of 77 patients [19]. In another cohort in Munich, elevated IL-6 (> 80 pg/ml) was strongly associated with a 22 times higher need for mechanical ventilation compared with patients with lower IL-6 levels in a cohort involving 40 patients, suggesting that high IL-6 level might predict the critical illness [20]. Another meta-analysis conducted by Muhammad et al. involved nine studies (1426 patients), and it confirmed that higher serum level of IL-6 was associated with increased risk of complicated COVID-19 and death [21], in which it suggested a cut-off value of 55 pg/ml. In accordance with these previous studies, we found that a cut-off of serum IL-6 (37.65 pg/ml) predicted death with high sensitivity and specificity.

In this cohort, we observed that the IL-6 levels were not necessarily decreased in the patients who are discharged or cured. This is quite different from the result reported by Gong et al*.*, which suggested that higher IL-6 levels in the disease course might indicate disease deterioration [13]. Our data appears in contrast with this suggestion since in our study after tocilizumab administration patients might have higher IL-6 levels than before. This phenomenon was also observed in the tocilizumab management of cytokine release syndrome induced by chimeric antigen receptor T (CAR-T) cell infusion, rheumatoid arthritis, and Castleman disease [22, 23]. The exact reason for the significant increase of serum IL-6 after administration of tocilizumab is still unknown. One of the potential explanations involves the restriction of receptor-bound IL-6 consumption [22]. Furthermore, a transient rise in IL-6 levels might increase the risk of CAR-T-cell-related encephalopathy syndrome [23]. Whether higher IL-6 levels resulted from administration of tocilizumab in COVID-19 patients with elevated baseline IL-6 will lead to central nervous system symptoms or other adverse events is worth further exploration, since tocilizumab might be difficult to penetrate the complete blood–brain barrier and then block the IL-6 signal in the brain.

Xu et al. reported the first results of tocilizumab treating COVID-19 in a retrospective study [10]. 20 patients were enrolled and after tocilizumab administration clinical symptoms and laboratory indicators were improved in most patients. Conrozier et al. reported a retrospective case series of 40 patients with COVID-19-acute respiratory distress syndrome (ARDS) treated with tocilizumab, in which 30 patients survived and 10 died [24]. Comparing with the case fatality rate of 22.8% (94/413) in all the patients with COVID-19 during the same period in their center, the authors suggested that tocilizumab result in favorable evolution in cases with COVID-19-ARDS. Similarly, in another retrospective case series in South Italy, IL-6 receptor antagonist sarilumab was prescribed in 15 patients with COVID-19 associated respiratory insufficiency. Rapid improvements in respiratory function as well as normalization of inflammatory markers were observed in 10 of 15 patients. The case fatality rate of this cohort was 33% [25]. Comparing to these previous studies, our cohort comprised more patients with moderate illness. Theoretically, using IL-6 receptor antagonist in early stage of COVID-19 might be more helpful to reverse the outcome. But tocilizumab failed to demonstrate its efficacy in regard to survival outcome under the examination of propensity-score matching design in our cohort. As a proinflammatory cytokine, IL-6 is pyrogenic. Blocking its receptor, it is predictable that body temperature might decrease, and respiratory distress might be relieved to some degree. However, we expect more than symptomatic treatment of tocilizumab in COVID-19. The survival outcome is pending until prospective, randomized, double-blind clinical trials can be performed.

This study has several limitations. First, this was a retrospective study, however single center might not be a disadvantage since the therapeutic strategy and laboratory tests might remove obvious heterogeneity amongst different centers. Second, due to the nature of compassionate use, characteristics were unbalanced in patients who received tocilizumab or did not receive it. Despite balancing with propensity-score match, unforeseen variables might be left unbalanced as well, which could compromise the rigidity of the results. Third, generally speaking, tocilizumab was administered in severe or critical patients, however, several common cases with moderate elevated IL-6 level received tocilizumab, which might impact the results. Besides, follow-up of respiratory functions as well as inflammatory biomarkers including C-reacting protein after tocilizumab treatment were inaccessible in this cohort, we fail to draw a formal conclusion regarding overall clinical benefit of tocilizumab.

## Conclusion

To our knowledge, this is the largest case series regarding the relationship between COVID-19 and serum IL-6 levels. This emerging disease still does not have an universally accepted severity stratification. We have confirmed the finding that serum IL-6 should be included in diagnostic work-up to stratify disease severity, but the benefit of tocilizumab needs further confirmation. In particular, the significance of remarkable increase in serum IL-6 after tocilizumab administration is worthy of further study.

## Supplementary information

**Additional file 1: Fig S1.** The kinetics of IL-6 concentrations in three severity subsets: common, severe, and critical COVID-19.

**Additional file 2: Table S1.** Characteristics and parameters of patients receiving tocilizumab administration.

## Data Availability

The datasets used and/or analyzed during the current study are available from the corresponding author (W.D.W.) on reasonable request.

## Abbreviations

IL-6: Interleukin-6
COVID-19: Coronavirus disease 2019
IQR: Interquartile range
AUC: Area under curve
CI: Confidence interval
SARS-CoV-2: Severe acute respiratory syndrome coronavirus 2
WHO: World Health Organization
IL-2R: Interleukin 2 receptor
IL-8: Interleukin 8
IL-10: Interleukin 10
TNFα: Tumor necrosis factor α
RT-PCR: Reverse transcription polymerase chain reaction assay
ROC: Receiver operator characteristic curve
CAR-T: Chimeric antigen receptor T
ARDS: Acute respiratory distress syndrome
