# Supplementary figures and images for: Serum interleukin-6 is an indicator for severity in 901 patients with SARS-CoV-2 infection: a cohort study
Source: J Transl Med. 2020 Oct 29;18:406. doi: 10.1186/s12967-020-02571-x (PMC7594951; doi:10.1186/s12967-020-02571-x)

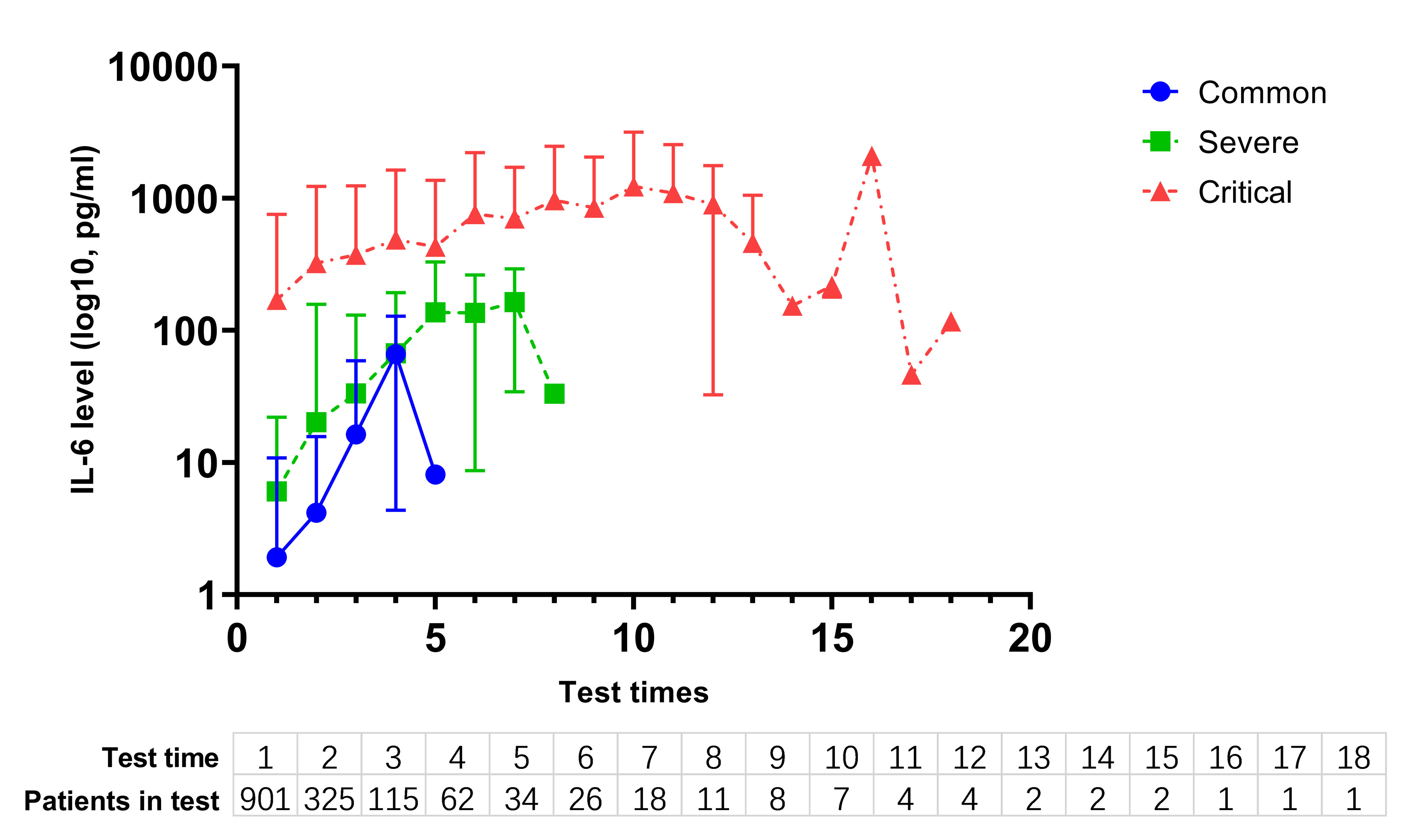

Supplement: Supplementary file 1 — Additional file 1: Fig S1. The kinetics of IL-6 concentrations in three severity subsets: common, severe, and critical COVID-19. [file 12967_2020_2571_MOESM1_ESM.jpg]
