# Supplementary material for: Serum interleukin-6 is an indicator for severity in 901 patients with SARS-CoV-2 infection: a cohort study
Source: J Transl Med. 2020 Oct 29;18:406. doi: 10.1186/s12967-020-02571-x (PMC7594951; doi:10.1186/s12967-020-02571-x)
Supplement: Supplementary file 2 — Additional file 2: Table S1. Characteristics and parameters of patients receiving tocilizumab administration. [file 12967_2020_2571_MOESM2_ESM.docx]

Table S1. Characteristics and parameters of patients received tocilizumab administration.

| **Patient ID.** | **Gender** | **Age** (years) | **Comorbidity** | **Severity^*^** | **IL-6 before tocilizumab** (pg/ml) | **Duration of IL-6**  **elevation** (days) | **Outcome^#^** |
| --- | --- | --- | --- | --- | --- | --- | --- |
| 18 | Male | 67 | W/O | Severe | 8.84 | 15 | Cured |
| 24 | Male | 64 | Hypertension | Common | 13.41 | 7 | Cured |
| 25 | Male | 68 | Hypertension, Pulmonary embolism, Hyperlipidemia, Cardiac pacemaker implantation history, Lower extremity arteriosclerosis occlusion stent implantation history | Critical | 146.90 | 26 | Hospitalization |
| 112 | Female | 85 | W/O | Severe | 14.57 | 16 | Improved |
| 113 | Female | 84 | N/A | Severe | 24.05 | 23 | N/A |
| 118 | Female | 79 | Coronary heart disease, Gastric disease | Severe | 25.11 | N/A | Cured |
| 119 | Male | 76 | Coronary heart disease, Hypertension, Renal insufficiency | Common | 13.71 | 5 | Cured |
| 241 | Female | 76 | W/O | Severe | 62.55 | 12 | Hospitalization |
| 272 | Male | 92 | W/O | Critical | 619.50 | N/A | Died |
| 368 | Female | 58 | Rheumatoid arthritis, Diabetes mellitus | Critical | N/A | 3 | Died |
| 430 | Male | 47 | Diffuse large B cell lymphoma, Hepatitis B, Diabetes mellitus | Critical | 252.20 | 12 | Died |
| 441 | Male | 64 | W/O | Severe | 16.38 | 24 | Hospitalization |
| 445 | Female | 69 | Diabetes mellitus | Severe | 31.25 | 26 | Cured |
| 626 | Male | 67 | Hypertension, Benign prostatic hyperplasia | Common | 7.32 | 15 | Cured |
| 675 | Female | 58 | Hypertension | Common | 10.39 | 18 | Cured |
| 798 | Female | 75 | Hypertension, Chronic gastritis, Schistosomiasis cirrhosis | Critical | 94.43 | 8 | Died |

^*^ According to the interim guideline of National Health Committee, PRC. The most severe stratification during individual hospitalization.

^#^ Last follow-up: the 2^nd^ April, 2020.

W/O: without; N/A: not available.
